# PW02-036 - Thermosensitive CA2+ assay innate immune cells

**DOI:** 10.1186/1546-0096-11-S1-A177

**Published:** 2013-11-08

**Authors:** T Remijn, M Stoffels, A Simon, JW van der Meer

**Affiliations:** 1 Department of General Internal Medicine, Radboud University Nijmegen Medical Centre, Netherlands; 2 Nijmegen Institute for Infection, Inflammation and Immunity (N4i), Netherlands; 3 Nijmegen Centre for Molecular Life Sciences, Nijmegen, Nijmegen, Netherlands

## Introduction

Temperature has immunomodulatory effects on innate immune cells: incubating monocytes at low temperatures for example increases pro-inflammatory responses after LPS-stimulation. The mechanisms mediating this effect are still unclear. The activity of several ion channels is sensitive to changes in temperature, leading to cytosolic calcium influx. Calcium is universally involved in cellular processes, its effects depending on the cellular context. Although not much is known about the role of calcium in immune cells, it has been shown that calcium is necessary for inflammasome assembly. Conventional methods of measuring calcium are not ideal to study the relation between temperature and intracellular calcium concentrations ([Ca^2+^]_i_).

## Objectives

In this study, we aimed to optimize a fluorescence-based assay to measure [Ca^2+^]_i_ in innate immune cells using a qPCR-machine in a 96-well format.

## Methods

[Ca^2+^]_i_ can be measured by loading cells with the indicator Fluo-4AM. However, its affinity (K_d_) is highly dependent on temperature. This is not established in detail because the indicator is mostly used in temperature independent assays. Here, we determined K_d_^Fluo-4^ for temperatures in a range of 5-55^o^C, enabling calculation of exact intracellular calcium concentrations. We applied the assay in different cell lines and primary human innate immune cell populations.

## Results

Exposing cells to decreasing temperature induces elevated intracellular calcium concentrations. Subsequently rising temperature restores [Ca^2+^]_i_ to basal levels. This [Ca^2+^]_i_ increase can be blocked by the aspecific ion channel antagonist Ruthenium Red.

## Conclusion

We show that the intracellular calcium concentration in immune cells increases significantly upon decreasing temperature. Blockade by Ruthenium Red indicates that this effect is mediated by thermosensitive ion channels. We successfully optimized a 96-well format fluorescense-based assay using a qPCR machine, enabling [Ca^2+^]_i_ measurements in innate immune cell populations under tight temperature control.

## Competing interests

None declared

